# Multiple Genetic Origins of Non‐Native, Self‐Sustaining Rainbow Trout *Oncorhynchus mykiss* in Streams in Baden‐Württemberg, Germany

**DOI:** 10.1111/eva.70271

**Published:** 2026-05-24

**Authors:** J. Peter Koene, Arne Jacobs, Patrick Bartolin, Jan Baer, David Frei, Pascal Vonlanthen, Alexander Brinker

**Affiliations:** ^1^ Scottish Centre for Ecology and the Natural Environment (SCENE) University of Glasgow Rowardennan UK; ^2^ School of Biodiversity, One Health and Veterinary Medicine University of Glasgow Glasgow UK; ^3^ Regierungspräsidium Karlsruhe, Fisheries Administration Karlsruhe Germany; ^4^ Fisheries Research Station of Baden‐Württemberg Langenargen Germany; ^5^ FORNAT AG Zürich Switzerland; ^6^ Amt für Natur, Jagd and Fischerei St. Gallen Switzerland; ^7^ Aquabios GmbH Cordast Switzerland; ^8^ University of Konstanz Constance Germany

**Keywords:** genomics, invasive species, naturalisation, salmonid, stocking

## Abstract

Introduced in the late 19th Century, *Oncorhynchus mykiss* (rainbow trout) has been stocked historically in streams throughout Baden‐Württemberg, Germany, and some populations have become self‐sustaining with unclear impact on native salmonid populations. We sampled 223 rainbow trout from 14 streams and 3 hatcheries, each with different levels of domestication, from which the streams are known to have been stocked. We conducted genomic analyses to uncover evidence confirming self‐sustaining populations, to deduce potential sources of these populations, to compare the genetic diversity of hatchery versus stream populations and to discover genetic differences between stream and hatchery populations. We found genetic population structuring amongst the stream populations, consistent with natural reproduction over several generations, and we inferred multiple genetic origins, potentially including source populations beyond the three hatcheries considered, indicating that naturalisation occurs independently of domestication level and that all lineages pose similar likelihoods of establishing in the wild. We found no significant difference in genetic diversity between stream and hatchery populations, but there were nine loci across four genomic regions associated with naturalisation within or adjacent to immunity, growth and development genes. Whether such genes are under selection in wild stream environments needs still to be determined to inform fisheries and conservation management.

## 1 Introduction

Since the late 19th Century, *Oncorhynchus mykiss* (Walbaum 1792) (rainbow trout), has been one of the most commonly introduced fish species worldwide (Halverson 2010). Both its popularity as a game fish and its favourable attributes for aquacultural food production—easy husbandry, tolerance of higher temperatures relative to other native salmonid species, easy manipulation of life history via selective breeding, to name a few—make it economically valuable (Crawford and Muir 2008; Stanković et al. 2015). Ultimately originating with a small number of source lineages from California in the 1870s, hatchery rainbow trout have been introduced to almost 100 countries, more than half of which now host established self‐sustaining populations in wild ecosystems (Stanković et al. 2015). Hatchery fish may be introduced to wild systems through deliberate stocking or as aquaculture escapees (Fausch 2007). When they naturalise (i.e., establish self‐sustaining populations) in the wild, they may become potentially invasive, even to the extent of inclusion as a fish example in the IUCN list of the 100 of the world's worst invasive alien species (Lowe et al. 2000). Their detrimental effects on native fish fauna in some regions are well documented: among others, predation upon native species, competition for space and resources and the introduction and transfer of novel pathogens (reviewed by Stanković et al. 2015). In Europe, there is particular concern over rainbow trout's impact on habitat selection and survival of native brown trout (*Salmo trutta*, L.) through competition, including redd superimposition (Blanchet et al. 2007; Landergren 1999). Interestingly, both rainbow trout and brown trout may be invasive when introduced to the native areas of other species, with brown trout more likely to displace native fish species (Gatz et al. 1987; Young et al. 2010). In places such as New Zealand, where both species are non‐native, brown trout appear to be more successful, even though spawning competition and redd superimposition there would seem to favour the later‐spawning rainbow trout (Hayes 1987). It has been established that adaptation to water chemistry variation related to underlying geology is a major driver of population structure in salmonid species, including brown trout. This may play a role in competitive success when salmonid species are co‐introduced to novel environments (Osmond et al. 2024; Paris et al. 2015). Generally, research is lacking on inverse competition in both species and the occurrence of rainbow trout in areas where brown trout are native (Fausch 2007; McGlade et al. 2022; Stanković et al. 2015).

However, serious declines in native European salmonid populations since the late 20th Century have led to questioning the prudence of rainbow trout stocking (Arlinghaus et al. 2002; Burkhardt‐Holm et al. 2002; Stanković et al. 2015), especially as stressors to native salmonids are exacerbated by effects of climate change (Ros et al. 2022; Rubin et al. 2022). In the federal state of Baden‐Württemberg in southern Germany, changes in management strategy have seen stocking of rainbow trout decline markedly and even be banned in some catchments to avoid competition with native brown trout (Baer and Brinker 2010). These bans were imposed in the 1990s as a precautionary measure, based mostly on findings from countries where neither brown nor rainbow trout is native (Hayes 1987). Yet, populations of rainbow trout persist in a small proportion of formerly stocked streams throughout the state (Stanković et al. 2015). It is known from oft‐documented information provided by fisheries managers (unpublished), and underlined by confirmation by fish farmers, that stocked trout originate from at least three commercial fish farms: one with a long history of domestication and advanced technological integration, a second with a medium history of domestication and medium degree of technological integration, and a third with a short history of domestication and low degree of technological integration. However, due to a paucity of records, it is not known from which of these farms the present naturalised populations stem. It is, therefore, also unknown whether descendants from all three hatchery lineages were able to establish themselves successfully in wild streams, or whether specific lineages were more likely than other lineages to naturalise and hence represent a greater potential danger to local fish fauna.

Naturalisation success of rainbow trout beyond its native range is thought to be more dependent upon genetic characteristics than environmental conditions (Koutsikos et al. 2019). It is not known, however, whether parallel naturalisation events resulted from specific highly invasive genetic lineages, or whether they proceed from multiple genomic origins which led to similar ends (Elmer and Meyer 2011). It has been suggested that multiple phenotypic traits known to have a strong genetic basis may play an important role in naturalisation success (Mueller et al. 2025): spawning time and egg size (Weber et al. 2023); ancestral anadromy versus riverine residency, which is underpinned by inversion in a large region of one chromosome, Omy5 (Pearse et al. 2014, 2019); and behavioural traits such as aggressiveness and activity level in the presence of native species (McGlade et al. 2022). Previous work on naturalised populations across European streams using mitochondrial DNA found high levels of genetic diversity compared to hatchery populations, which may be crucial for naturalisation success (Stanković et al. 2016). However, to our knowledge, the greater resolution of interpopulation relationships between self‐sustaining rainbow trout and their aquacultural sources in Europe offered by studies that span the genome have not yet been conducted.

We analysed population structure across hatchery and stream populations in southern Germany through restriction‐site‐associated sequencing (RADseq) of genomic DNA to determine whether genomic data is consistent with natural reproduction across the study streams, to deduce the sources of naturalised populations, to compare the genetic diversity of hatchery vs stream populations, and to discover genetic differences, including regions of genomic differentiation, between stream and hatchery populations.

## 2 Methods

### 2.1 Sampling, Sequencing and Data Processing

**FIGURE 1:**
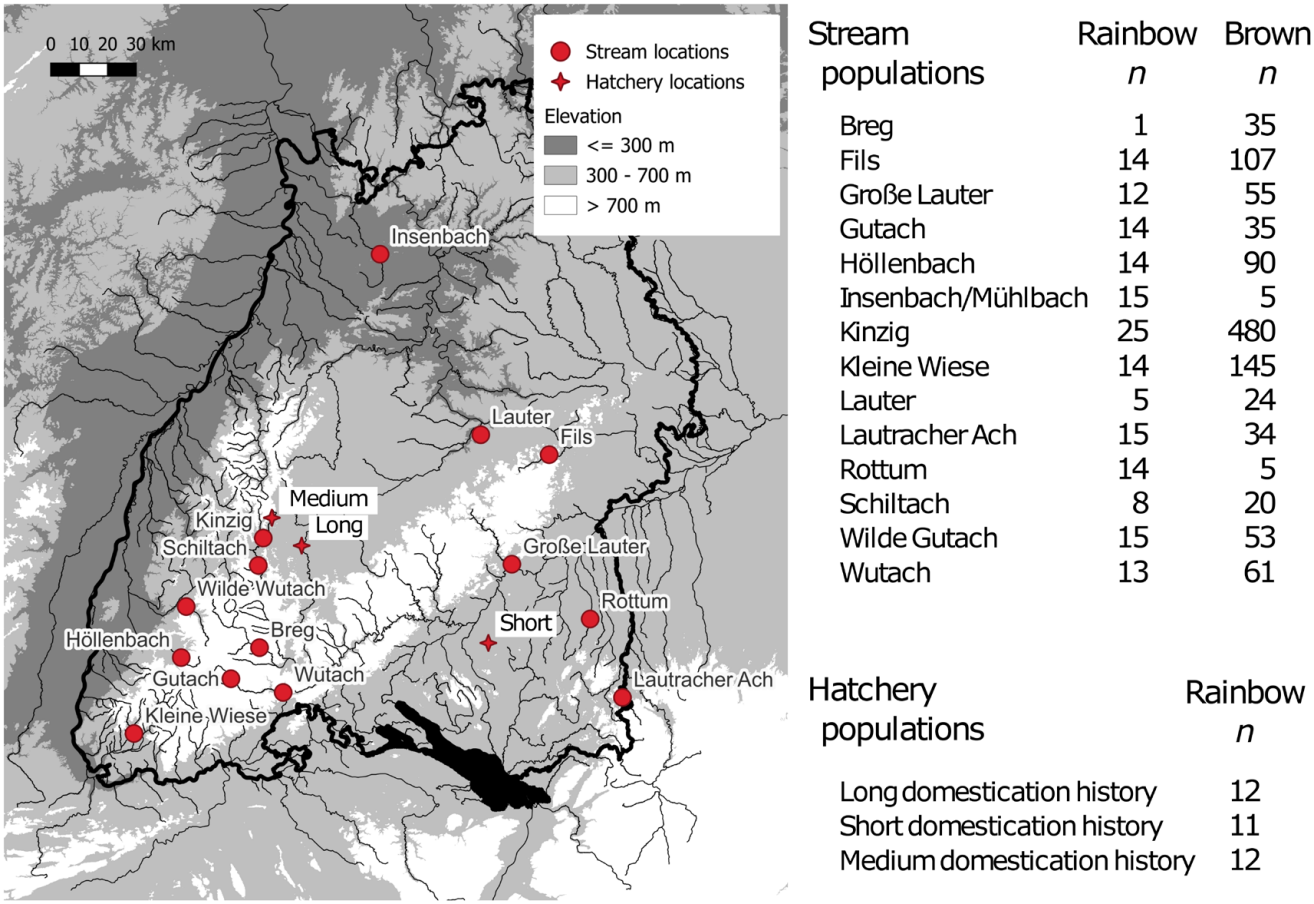
Sampling locations of 14 stream populations of rainbow trout and brown trout, and 3 rainbow trout hatcheries in Baden‐Württemberg, Germany, with numbers of specimens obtained from each location.

The database of the Fisheries Research Station of Baden‐Württemberg, which contains data on fish stock surveys in the waters of the German federal state of Baden‐Württemberg from the past 40 years (< 80,000 datasets), was searched for potential self‐reproducing rainbow trout stocks. In addition, local experts were asked whether they knew of any further stocks of naturally reproducing rainbow trout. Subsequently, rainbow trout specimens (*n* = 219) from proven self‐sustaining populations in 14 wild streams across Baden‐Württemberg were captured by a combination of electrofishing (single anode, 600 V DC electrofishing setup, 0.65 kW, Bretschneider Spezialelektronik) and angling (fly fishing, dry fly or nymph) (Figure 1). That the populations were self‐sustaining was demonstrated by the cessation of stocking in each of the 14 streams around the 1990s (Baer and Brinker 2010), and the observed presence of young‐of‐year, spawning redds and active spawning. Brown trout caught at the same time using the same methods were counted for comparison. All fish were caught by licensed personnel under permission of the local fisheries administration (Regierungspräsidium Karlsruhe, Freiburg, Tübingen) according to the German Animal Protection Law (§ 4). Captured rainbow trout were killed according to the ordinance on slaughter and killing of animals (Tierschutzschlachtverordnung § 13). Additionally, rainbow trout specimens were sampled from three aquaculture facilities that had previously been used to stock the sampled streams. Those rainbow trout have undergone domestication for up to a century as the products of captive brood lines, selected almost exclusively from within their own populations, across multiple generations. Fin clips were immediately taken from all specimens after euthanasia, stored in analytical ethanol and shipped to Floragenex Inc. (USA) for genomic DNA extraction, normalisation to 20 ng/μL, paired‐end 150 bp library preparation and sequencing with an Illumina Novaseq S4 flow cell on the Illumina Novaseq 6000 platform.

Quality of raw data was checked using *FastQC v.0.12.0* (https://www.bioinformatics.babraham.ac.uk/projects/fastqc/). Reads were assigned to individual samples, and duplicates removed, using *process_radtags* 2.4 and *clone_filter* 2.4, respectively, within *Stacks* (Catchen et al. 2013). Reads were aligned to the rainbow trout reference genome (Pearse et al. 2019; GenBank Assembly Accession GCA_013265735.3 USDA_OmykA_1.12020) using BWA‐MEM (Li 2013). Conversion of sam to bam files was made with *sambamba v.1.0* (Tarasov et al. 2015), and *angsd v.0.941* (Korneliussen et al. 2014) was used to calculate genotype likelihoods and estimate population genomic summary statistics. Genotype likelihoods allow use of more of the sequencing data across the genome than would be possible with explicit genotype calling, and they account for the uncertainty in genotype calls (Shafer et al. 2017). A range of common read‐specific and site‐specific filters was applied during the initial SNP calling step in *angsd* to retain only highly supported sites for downstream analyses (Akopyan et al. 2026; Lou and Therkildsen 2022). First, reads were excluded if they had a mapping quality lower than 30 (‐minMApq 30), a flag above 255 (‐remove_bads 1), were not properly paired (‐only proper pairs 1); and mapping quality was adjusted for excessive matches (‐C 50). Second, individual positions were only considered if the base quality score was above 20 (minQ 20), if at least half of all individuals had two reads, the minimum read depth was above 1 × the total number of individuals (min depth of 219) across all individuals, and the maximum read depth was below 2 × mean depth × total number of individuals (maximum depth of 8353). Lastly, positions were considered single nucleotide polymorphisms (SNPs) if the SNP *p*‐value for the likelihood that a site is variable was below 10e^−6^ and sites had two alleles (‐skipTriallelic 1). SNPs with a rarer allele frequency of less than 5% were further excluded.

### 2.2 Analyses

All analyses were, unless otherwise indicated, conducted with *R v.4.5.1* (R Core Team 2025). A principal components analysis (PCA) based on identified SNPs after filtering was conducted using *PCAngsd v.1.2* (Meisner and Albrechtsen 2018). The same package was used to construct admixture plots showing ancestry proportions for all hatchery and stream individuals. *PCAngsd* automatically infers K (i.e., the likely number of genetic clusters that best explain genetic structure) as the number of inferred principal components plus 1. The fit of ancestry models was further evaluated using correlations of residuals in *evalAdmix* for *K* = 2 to *K* = 16 (Garcia‐Erill and Albrechtsen 2020). A phylogenetic tree was constructed following the neighbour‐joining method, based on covariance matrices inferred with *PCAngsd*, to visualise relationships between populations using *FigTree v.1.4.4* (http://tree.bio.ed.ac.uk/software/figtree/).

A second genomic PCA was performed in *PCAngsd v.1.2* (Meisner and Albrechtsen 2018) for SNPs within the Omy5 region alone, where an inversion has previously been described (Pearse et al. 2019): chromosome Omy5, positions 35,000,000–85,000,000. All individuals were assigned to one of three possible genotypes of the Omy5 inversion, based on their genetic clustering in the PCA for this region (Figure 3A), with individuals in the middle cluster being classified as heterozygous for the inversion. Genotype frequencies (homozygous for the major allele, homozygous for the alternative allele, and heterozygous) were calculated for each population and origin type (i.e., hatchery vs. stream).

Genetic diversity values, that is, nucleotide diversity (*π*) and Watterson's *θ* (*θ*_w_), were calculated with *angsd* by population in 100 kb non‐overlapping windows across the genome using all sites, variant and invariant. Included sites were only those that were present in at least 50% of individuals per population, had a minimum coverage of 2× per included individual, a maximum coverage of 2 × mean depth (i.e., 18.8×) × number of individuals, and passed all other basic filters (i.e., mean mapping quality of 30, mean base quality of 20), but without filtering for minor allele frequency. These filters were applied to individual populations and genetic diversity values were estimated from the per‐population saf using the realSFS and thetaStat tools in *angsd*. A linear mixed effects model was fitted with the lmer function in the *R*‐package, *lme4* (Bates et al. 2015), to test the effect of origin (hatchery vs stream) on *π* and *θ*_w_. To control for interpopulation variation in both hatchery and stream groups, specific population was treated as a random factor.

Genetic differentiation (*F*_ST_) was calculated across all filtered SNPs using *angsd* with the realSFS tool (Korneliussen et al. 2014) and visualised with a Manhattan plot created in *CMplot v.4.2.0* (Yin et al. 2021). First, pairwise comparisons of *F*_ST_ values were made to establish means between hatchery–hatchery, stream–hatchery, and stream–stream populations. Second, *F*_ST_ was estimated on a per‐SNP basis and in 100 kb sliding windows with 25 kb steps between combined hatchery populations and all combined stream populations. Differences in genome‐wide *F*_ST_ between pairwise comparisons were tested with ANOVA followed by Tukey's HSD *post hoc*.

Finally, we conducted a genome‐wide association study (GWAS) to determine positions in the genome that are associated with origin (hatchery or stream). We created a mean genotype file from genotype probabilities and accounted for genetic covariance in a linear mixed model, using the lmm1 command in *GEMMA v.0.98.5* (Zhou and Stephens 2012) and visualised results with a Manhattan plot. We determined loci as significant if the *p*‐value was below a conservative threshold of 0.00000001 (10^−8^), which is well established for common variants and takes multiple testing into account (Fadista et al. 2016). Loci showing a significant association were manually checked against the rainbow trout genome using the genome browser, *Salmobase* (https://salmobase.org), to identify genes within which the SNPs were found plus adjacent genes.

## 3 Results

### 3.1 Sampling

Rainbow trout were present in all 14 streams surveyed, although in some locations in very low numbers (i.e., Breg, Lauter and Schiltach; Figure 1). An object of particular management concern, native brown trout were also present in each stream, mostly either in numbers comparable with rainbow trout, or at densities many times higher. Only in Insenbach/Mühlbach and Rottum did rainbow trout numbers exceed those of brown trout (i.e., 15:5 and 14:5 respectively; Figure 1).

### 3.2 Population Structure

**FIGURE 2:**
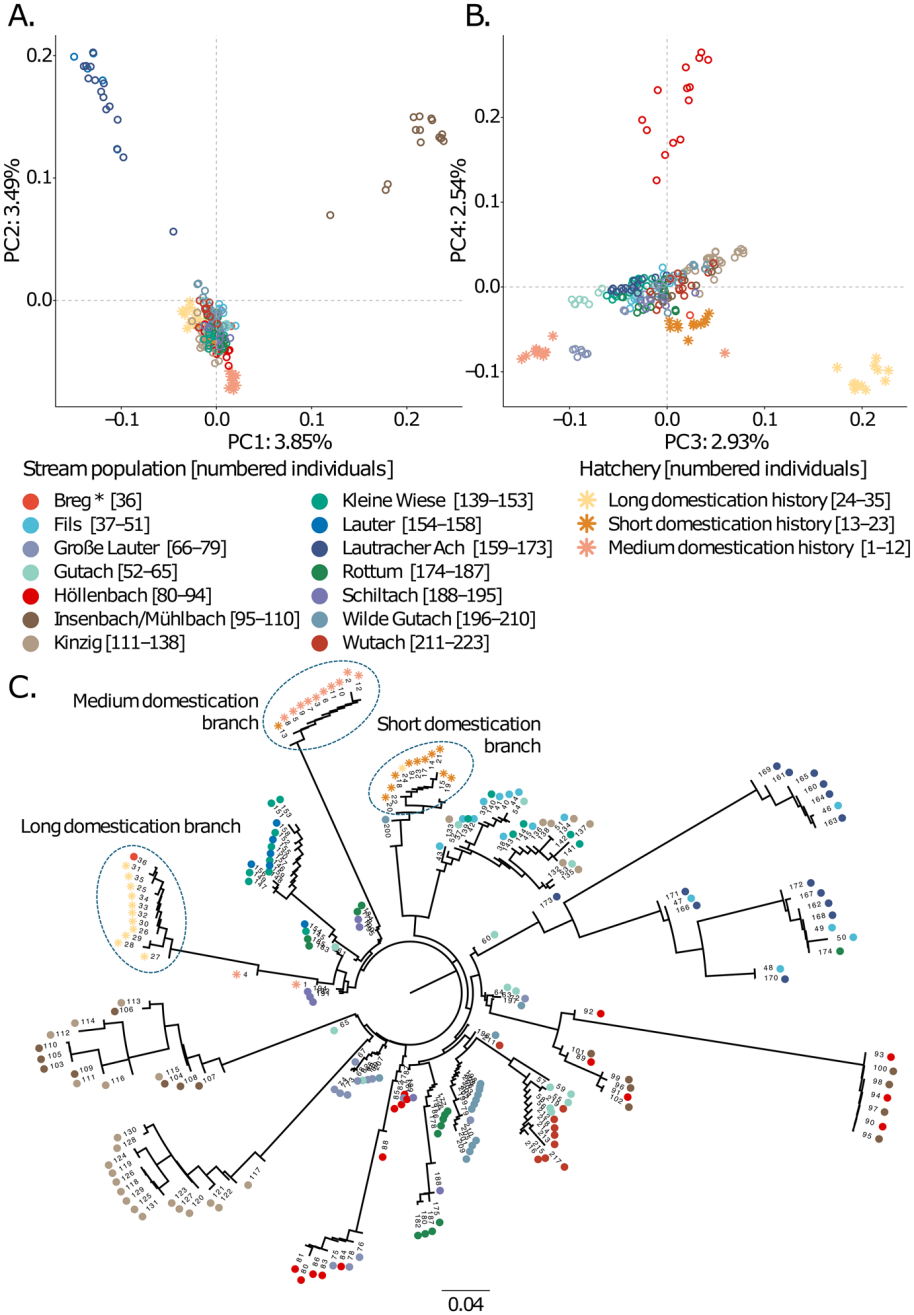
Population structure of 3 hatchery and 14 stream populations of rainbow trout, displayed as (A) PC1 and PC2 axes, and (B) PC3 and PC4 axes of a genomic PCA; and (C) a phylogenetic tree constructed by neighbour‐joining method, in which the main hatchery branches are specifically marked. * Nb. Breg is represented by a single individual.

After filtering, a total of 533,536 SNPs with a mean depth of coverage of ~18× were identified. The PCA based on SNPs revealed three main clusters on the PC1 and PC2 axes, representing 3.85% and 3.49% of variation explained, respectively: one containing only the Insenbach/Mühlbach population, another containing Lauter and Lautracher Ach populations, and the third containing the remaining naturalised populations together with hatchery populations (Figure 2A). Within the large third PCA cluster, individuals from the hatchery with the shortest domestication history stood slightly apart from the remaining populations. On the PC3 and PC4 axes, representing 2.93% and 2.54% of variation explained, respectively, the three hatcheries were separated clearly from one another, while the Höllenbach stream population clustered on its own, and there was further fine scale clustering amongst several other populations (Figure 2B). These population structuring patterns were confirmed by a neighbour‐joining phylogenetic tree (Figure 2C), which showed branches dominated by populations, but with some mixture of populations on most major branches; and the admixture analysis, which showed clear correspondence of the 15 genetic clusters that best explained structure with population, although admixture was widespread (Figures S1 and S2). The pairwise correlation of residuals in the admixture model was also minimised at a K of 15 the *evalAdmix* analyses, suggesting that this model is a good approximation of ancestry clusters.

**FIGURE 3:**
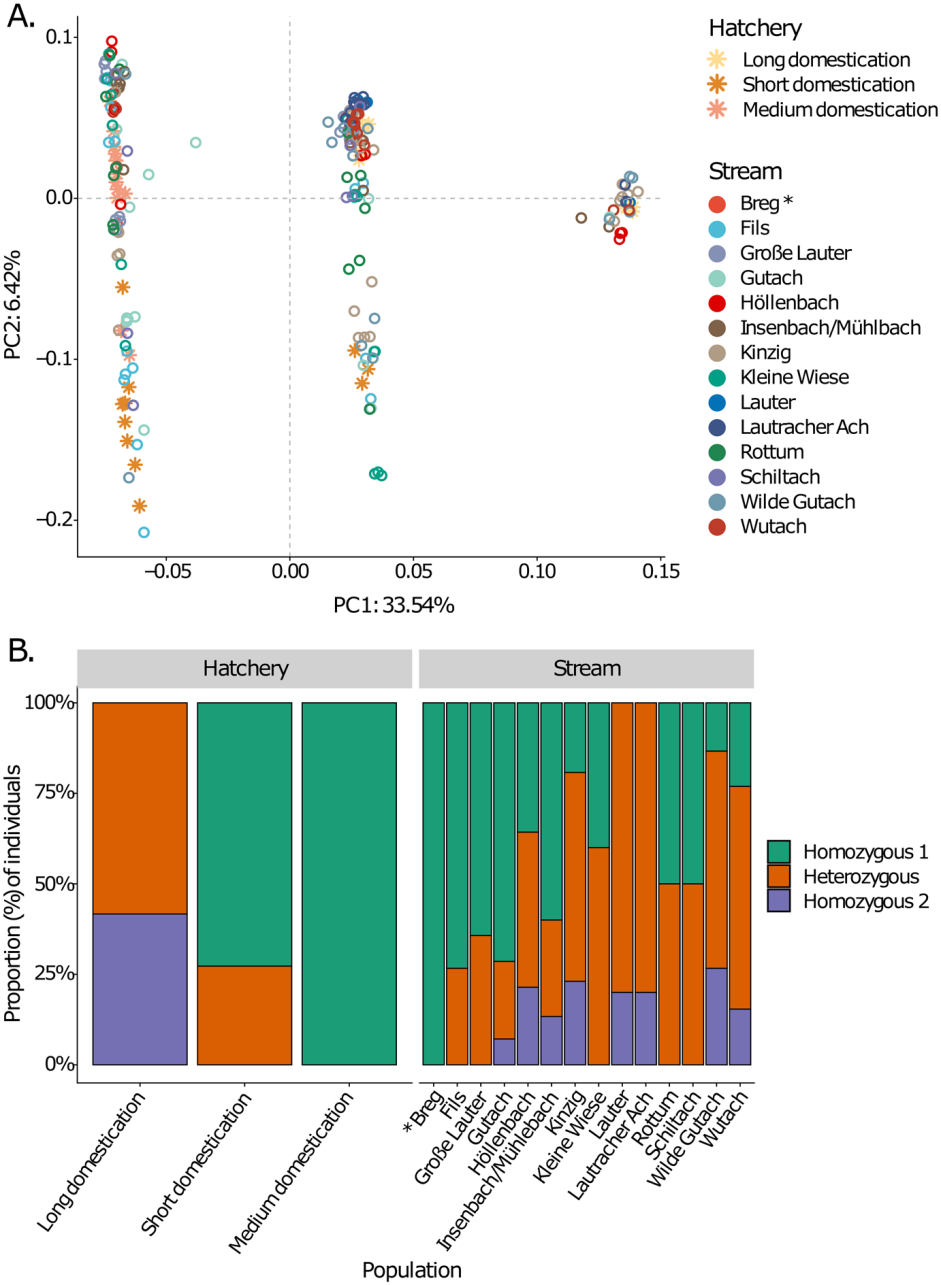
Variation in the Omy5 chromosome across 3 hatchery and 14 stream populations of rainbow trout: (A) clustering in PCA; (B) proportion of individuals by population homozygous with the major allele, alternate allele or heterozygous. * Nb. Breg is represented by a single individual.

Regarding the Omy5 inversion, which was chosen for particular consideration because of its association with life history variants, three distinct clusters showed clearly on the PC1 axis, explaining a large proportion (33.5%) of genetic variation in this region (Figure 3A). The pattern corresponds to three genotype variants, which were assumed to represent the three inversion types: homozygous for the major allele, homozygous for the alternative allele and heterozygous. All three genotypes were present in both hatchery and stream populations, without any clear difference in inversion frequencies between hatchery and stream populations (Figure 3B).

### 3.3 Genetic Diversity

Although we found variation between individual populations, there was no significant difference in genetic diversity across the genome, generally, between hatchery and stream trout: the effect of origin (stream) on *π* was β = 2.43e^−4^, 95% CI [−5.4e^−5^, 5.4e^−4^], t_297695_ = 1.6, *p* = 0.109; Std. β = 0.13, 95% CI [−0.03, 0.29]; the effect of origin (stream) on *θ*_w_ was β = 2.66e^−4^, 95% CI [−1.01e^−4^, 6.34e^−4^], t_297695_ = 1.42, *p* = 0.156; Std. β = 0.18, 95% CI [−0.07, 0.42] (Figures S3 and S4).

### 3.4 Genetic Differentiation

**FIGURE 4:**
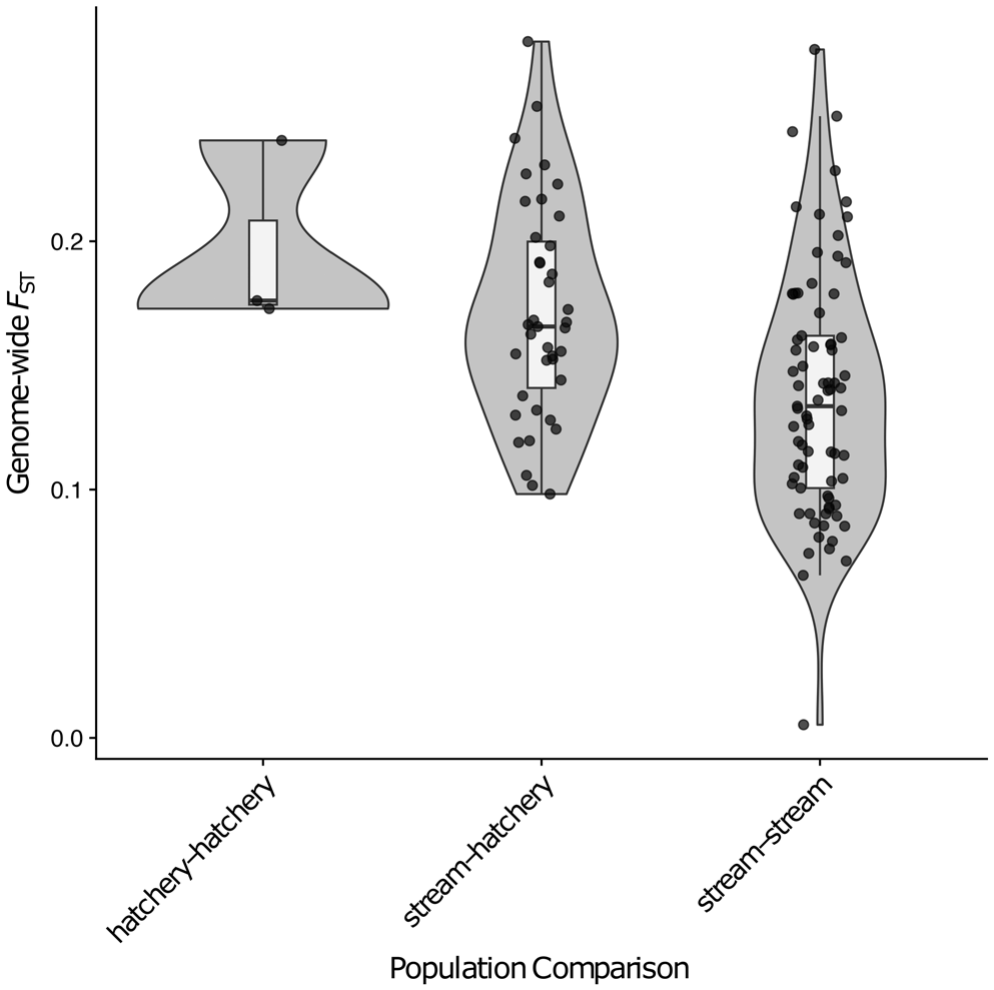
Effect of comparison on weighted *F*_ST_ across the genome. Comparisons were made between 3 hatchery populations, 14 stream populations and hatchery and stream populations of rainbow trout.

**FIGURE 5:**
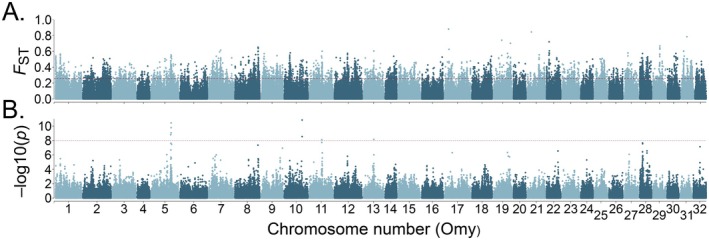
Manhattan plots showing (A) genetic differentiation (*F*_ST_) across the genome, and (B) values of −log(10) of *p* from a genome‐wide association study of 3 hatchery and 14 stream populations of rainbow trout. Chromosomes are coloured in alternating order for visual clarity. The dashed red line in (A) shows the 99th percentile of the genome *F*_ST_ distribution (*F*_ST_ = 0.259) and the dashed red line in (B) shows the *p*‐value threshold for significant genome‐wide estimation (*p* = 0.00000001).

Genetic differentiation was pronounced for all population comparisons (Figure 4; Figure S5), with the greatest distinction between hatchery populations (mean *F*_ST_ ± s.d. = 0.1965 ± 0.0382), followed by stream–hatchery comparisons (mean *F*_ST_ ± s.d. = 0.1715 ± 0.0437), and the least differentiation between stream populations (mean *F*_ST_ ± s.d. = 0.1377 ± 0.049). There was a significant effect of comparison on weighted *F*_ST_ (*F*_2,116_ = 8.11, *p* < 0.001), with the difference between the stream–stream comparison significantly lower than the stream–hatchery comparison (difference = −0.0337, 95% CI [−0.0557, −0.0117], *p* = 0.001) (Figure 4). However, there was no strong signal of differentiation across the Omy5 inversion (Figure 5A; Figure S6), as suggested by Omy5 inversion frequencies in individual hatchery and stream populations. We detected widespread genetic differentiation across the genome between hatchery and stream populations with genomic regions on most chromosomes showing differentiation above the 99th percentile of the *F*_ST_ distribution (*F*_ST_ > 0.259). Between individual populations, Insenbach/Mühlbach was most differentiated from the hatcheries (from long domestication, *F*_ST_ = 0.2804, from short domestication, *F*_ST_ = 0.2271, and from medium domestication, *F*_ST_ = 0.2543), followed by Lauter (from long domestication, *F*_ST_ = 0.2415, from short domestication, *F*_ST_ = 0.1916, and from medium domestication, *F*_ST_ = 0.2308), and Lautracher Ach (from long domestication, *F*_ST_ = 0.2170, from short domestication, *F*_ST_ = 0.1725, and from medium domestication, *F*_ST_ = 0.2101). *F*_ST_ values between Insenbach/Mühlbach and both Lauter and Lautracher Ach were larger than average (*F*_ST_ = 0.2772 and 0.2504, respectively). There was, however, little difference between Lauter and Lautracher Ach (*F*_ST_ = 0.0053).

### 3.5 Genome‐Wide Association With Origin

The GWAS identified nine SNPs significantly associated with origin (hatchery vs. stream), four of which were found within a narrow peak inside the Omy5 inversion. The other SNPs were located on chromosomes Omy10, Omy11 and Omy13. These origin‐associated regions also showed increased genetic differentiation between hatchery and stream populations. Significant GWAS SNPs were located within or close to genes encoding for proteins involved in amino acid and protein transport; phosphatase activity; development and proliferation of T‐cells; neural and cardiac development; and cell growth, proliferation, homeostasis and apoptosis (Figure 5B; Table S1).

## 4 Discussion

Population structuring that shows mixed ancestry amongst the rainbow trout populations in the examined streams of Baden‐Württemberg, without substantial loss of genetic diversity, and in the absence of continued stocking, is generally consistent with the assumption that these populations reproduce naturally and are self‐sustaining: although most populations appear to be related, structuring is present throughout the region. Patterns of genetic differentiation are indicative of drift over generations. Most of the stream populations showed ancestry from all three hatchery populations considered here, suggesting that these hatcheries were indeed source populations for most of the streams, with only Lauter and Lautracher Ach as exceptions. This is further supported by the lower genetic differentiation between stream populations compared to the hatchery populations, which might suggest a mixed hatchery origin of stream populations, potentially through genetic mixing caused by multiple stocking events or post‐colonisation gene flow between streams. A lack of connectivity between streams renders post‐colonisation gene flow highly unlikely, however. A reduction in genetic diversity (as opposed to differentiation) is a commonly recognised signature of founder events, in which only a portion of an original source population's genetic diversity is carried to a novel environment; however, the loss can be mitigated by the number of colonisation events and sources (Barton and Charlesworth 1984; Roman 2006). In comparing the stream rainbow trout to the hatchery populations, no evidence was found of reduced genetic diversity, further suggesting that multiple sources contributed to their naturalisation (Lázari et al. 2024). It is possible that prolonged stocking has provided sufficient subsamples from source hatcheries, or that there have been single inputs large enough to maintain genetic diversity. However, the substantial admixture found in the stream populations along with evidence from other lines of inquiry points to multiple sources.

Three stream populations stood out as particularly distinct both from other stream populations and the hatcheries. However, the mechanisms of differentiation in the populations at Insenbach/Mühlbach, Lauter and Lautracher Ach can only be subject to speculation. One possibility is that these populations have arisen from other breeding lineages of different geographic origin or hatcheries not considered here—potentially additional source lineages, as indications are that the Lauter and Lautracher Ach populations share a common ancestry. It may be that differing local environmental conditions in individual streams may present some unidentified selection pressure that promotes adaptation, which can occur over relatively few generations (Schluter 2009), and has been documented after historical stocking events in salmonids (e.g., Crotti et al. 2021; Koene et al. 2019; Vuorinen et al. 1991). However, population structuring based primarily upon presumably neutral loci would argue against adaptation. It is also possible that the differentiation of these three populations results from founder effects of a small historical stocking input followed by genetic drift (Hauser et al. 1995; Weeder et al. 2005), as has been described in other rainbow trout naturalisation events (Colihueque et al. 2019), although the genetic diversity of the stream populations would appear to argue against this (Austerlitz et al. 1997). Comparisons to other putative source populations would be required to distinguish between scenarios; however, we are unaware of any other potential source populations, and so were unable to include these in the study.

Three genotypes of the Omy5 inversion associated with life history were present amongst the stream populations, potentially corresponding to the homozygous anadromous, homozygous resident and heterozygous genotypes described in the literature (Goetz et al. 2024; Pearse et al. 2014), although the association of Omy5 genotype and life history is not always direct (e.g., Weinstein et al. 2019). Nonetheless, we found no strong *F*_ST_ signal within the Omy5 inversion. The frequencies of genotypes were not consistent across either hatchery or stream populations, and it is therefore not possible to evaluate the role that the inversion plays in the naturalisation of rainbow trout in wild streams.

On the other hand, of the nine positions identified by the GWAS to be associated with naturalisation, four were found within the Omy5 inversion. These positions were found within, or adjacent to, genes associated with immunity and growth and development, which are presumably under different selection pressures in the wild compared to hatchery environments, and may be related to phenotypes conducive to naturalisation (Kanno et al. 2017; Lázari et al. 2024). Consistent with our hypothesis that genetics play a role in determining whether rainbow trout can naturalise in wild ecosystems, we found that certain genetic variants are more likely than others to become established in the wild, which points to potential drivers of adaptation. It has been stated that the successful establishment of self‐sustaining rainbow trout populations is driven primarily by genetic factors (Koutsikos et al. 2019), but that environmental conditions play important roles, too (Fausch et al. 2001); we cannot rule out the possible importance of environmental variables. Furthermore, we found no evidence that the descendants of any one of the three hatchery populations had become more prevalent than the others in the wild, thereby posing a particular risk of spreading an invasive species. However, we cannot discount the possibility of a prolific source population, which has simply escaped our notice.

Our finding that the genetic profiles of all sampled hatchery stock are broadly represented in the rainbow trout of the different streams has important implications for the management of local trout waters, as the source of the stocking material appears not to be as important as hypothesised for the future establishment of rainbow trout stock. Because different stocked trout had come from hatchery stocks intensively selected for aquaculture optimisations, we hypothesised that the longer the selection history and/or the more artificial the rearing environment, the smaller the chance of establishment in the wild would be (Petersson et al. 1996; Lorenzen et al. 2012). However, our data do not support this hypothesis, as the longest, shortest and medium domesticated lineages were all represented in naturalised populations, without one hatchery predominant. Current levels of domestication or husbandry practice, at least, do not appear to prevent naturalisation probabilities: regardless of stocking source or time span of domestication, all stocked rainbow trout appear to have the potential to establish in nature. As a caveat, it must be considered that precisely when each population established in the wild is unrecorded; therefore, the state of domestication at the time of stock introduction and naturalisation cannot be known. Additionally, we must acknowledge that we sampled only three different farms with distinct stock characteristics, and that further investigation is needed for a more in‐depth understanding of the properties important for naturalisation.

Furthermore, the intensity of postulated competition with native brown trout (Stanković et al. 2016) has to be questioned. Despite more than 100 years of intensive rainbow trout stocking in southwestern Germany, extensive research was required to determine the areas in which reproducing populations occur. Although many, possibly even all, trout streams in Baden‐Württemberg had been increasingly stocked with rainbow trout over the past century, in only a few places have rainbow trout been able to establish self‐sustaining populations. Additionally, we found brown trout still present in all streams, where they co‐exist with rainbow trout. Rainbow trout were never dominant; indeed, often it was the brown trout populations that predominated. In many streams, rainbow trout establishment has occurred at very low levels, and in some instances with no apparent signs of local population expansion (i.e., increase in density or range) in more than 20 years, nor with signs of negative effects on brown trout populations (Baer and Brinker 2010). Therefore, the widely postulated phenomenon of competition, based mainly on studies from countries where both species are allochthonous (Hayes 1987), appears to exist only to a limited extent, at most, in the investigated ecoregion. However, some recent facilitation dynamic is evident, as documentation of self‐sustaining rainbow trout populations is limited to recent decades, despite more than a century of intense stocking. This study indicates that the propagule pressure framework, which relates invader establishment success to the density of introduced individuals, the number of introduction events and the relative frequency of introductions (Simberloff 2009), does not adequately explain the patterns observed in this species and ecoregion. Instead, the invasion potential of rainbow trout has likely increased recently through, for example, the emergence of additional invasive species and/or the effects of climate change. Further research is therefore required to identify the conditions under which rainbow trout can successfully naturalise, and to quantify any resulting impacts on local brown trout populations.

In summary, we found clear population structuring of rainbow trout populations in the streams of Baden‐Württemberg, which provided evidence of natural reproduction over several generations. Multiple genetic origins of stream populations were inferred: the three hatcheries under consideration here, and possibly others; and the origins were likely mixed via pre‐ or post‐colonisation gene flow. However, despite multiple origins, there was no significant difference in genetic diversity between stream and hatchery populations, but there were specific positions in the genome associated with naturalisation. Fisheries managers will benefit from more detailed knowledge of the locations, comparative abundance, population structure and origins of naturalised rainbow trout in their assessments of potential threats to native salmonids.

## Funding

This project was funded by the Fischereiabgabe Baden‐Württemberg.

## Ethics Statement

All fish were caught by licensed personnel under permission of the local fisheries administration (Regierungspräsidium Karlsruhe, Freiburg, Tübingen) according to the German Animal Protection Law (§ 4). Captured fish were killed according to the ordinance on slaughter and killing of animals (Tierschutzschlachtverordnung § 13).

## Conflicts of Interest

The authors declare no conflicts of interest.

## Supporting information


**Figure S1:** Admixture analysis across 3 hatchery and 14 wild stream populations of rainbow trout. Genetic structure was best explained with 15 genetic clusters (*K* = 15), based on the number of principal components in *PCAngsd* and the correlation of residuals (*vide* Figure S2, below). *Nb. Breg is represented by a single individual.
**Figure S2:**. EvalAdmix results. Shown are correlations of residuals for each pairwise comparison of individuals/populations. Correlations were inferred with evalAdmix for ancestry proportions in Figure S1 and were used to select the value of K at which correlations of residuals are minimised (closest to 0) (*K* = 15). Cells above the diagonal are individual‐specific, and cells below the diagonal are averaged by population. Population and ecotype labels are shown next to each group. *Nb. Breg is represented by a single individual.
**Figure S3:**. Genetic diversity measured as *π* in 100 kb windows across the genome of 3 hatchery and 13 stream populations of rainbow trout. One stream population (Breg) was omitted because it was represented by a single individual. Outliers are included in (A), and excluded in (B).
**Figure S4:**. Genetic diversity measured as Watterson's *θ* in 100 kb windows across the genome of 3 hatchery and 13 stream populations of rainbow trout. One stream population (Breg) was omitted because it was represented by a single individual. Outliers are included in (A), and excluded in (B).
**Figure S5:**. Pairwise weighted F_ST_ heatmap of 120 comparisons across 16 populations of rainbow trout. Dashed lines separate hatchery (red) from wild stream populations.
**Figure S6:**. Genome‐wide differentiation between hatchery and stream populations in 100 kb sliding windows and 25 kb steps. Slightly broader peaks of differentiation (e.g., on chr28) also show similar signals in the SNP‐by‐SNP comparison (Figure 4A).

## Data Availability

All environmental and ecological data are included within either this article or its Appendix S1. Sequence reads are archived at the European Nucleotide Archive (ENA) under project accession PRJEB113258.

## References

[eva70271-bib-0001] Akopyan, M. , A. Jacobs , J. A. Rick , et al. 2026. “Multiple Chromosomal Inversions Modulate Continuous Local Adaptation Along a Steep Thermal Cline.” Science 391, no. 6789: 1015–1021. 10.1126/science.ady6774. 41785359

[eva70271-bib-0002] Arlinghaus, R. , T. Mehner , and I. G. Cowx . 2002. “Reconciling Traditional Inland Fisheries Management and Sustainability in Industrialised Countries, With an Emphasis on Europe.” Fish and Fisheries 3, no. 4: 261–316. 10.1046/j.1467-2979.2002.00102.x.

[eva70271-bib-0003] Austerlitz, F. , B. Jung‐Muller , B. Godelle , and P.‐H. Gouyon . 1997. “Evolution of Coalescence Times, Genetic Diversity and Structure During Colonisation.” Theoretical Population Biology 51, no. 2: 148–164. 10.1006/tpbi.1997.1302.

[eva70271-bib-0004] Baer, J. , and A. Brinker . 2010. “The Response of a Brown Trout Stocks and Perception of Anglers to Cessation of Brown Trout Stocking.” Fisheries Management and Ecology 17, no. 2: 157–164. 10.1111/j.1365-2400.2009.00713.x.

[eva70271-bib-0005] Barton, N. H. , and B. Charlesworth . 1984. “Genetic Revolutions, Founder Effects, and Speciation.” Annual Review of Ecology and Systematics 15: 133–164. 10.1146/annurev.es.15.110184.001025.

[eva70271-bib-0006] Bates, D. , M. Mächler , B. Bolker , and S. Walker . 2015. “Fitting Linear Mixed‐Effects Models Using lme4.” Journal of Statistical Software 67: 1–48. 10.18637/jss.v067.i01.

[eva70271-bib-0007] Blanchet, S. , G. Loot , G. Grenouillet , and S. Brosse . 2007. “Competitive Interactions Between Native and Exotic Salmonids: A Combined Field and Laboratory Demonstration.” Ecology of Freshwater Fish 16, no. 2: 133–143. 10.1111/j.1600-0633.2006.00205.x.

[eva70271-bib-0008] Burkhardt‐Holm, P. , A. Peter , and A. H. Segner . 2002. “Decline of Fish Catch in Switzerland.” Aquatic Sciences 64: 36–54. 10.1007/s00027-002-8053-1.

[eva70271-bib-0009] Catchen, J. , P. A. Hohenlohe , S. Bassham , A. Amores , and W. A. Cresko . 2013. “Stacks: An Analysis Tool Set for Population Genomics.” Molecular Ecology 22, no. 11: 3124–3140. 10.1111/mec.12354. 23701397 PMC3936987

[eva70271-bib-0010] Colihueque, N. , F. J. Estay , J. E. Crespo , et al. 2019. “Genetic Differentiation and Origin of Naturalised Rainbow Trout Populations From Southern Chile, Revealed by the mtDNA Control Region Marker.” Frontiers in Genetics 10: 1212. 10.3389/fgene.2019.01212. 31921284 PMC6933019

[eva70271-bib-0011] Crawford, S. S. , and A. M. Muir . 2008. “Global Introductions of Salmon and Trout in the Genus *Oncorhynchus*: 1870–2007.” Reviews in Fish Biology and Fisheries 18: 313–344. 10.1007/s11160-007-9079-1.

[eva70271-bib-0012] Crotti, M. , E. Yohannes , I. J. Winfield , A. A. Lyle , C. E. Adams , and K. R. Elmer . 2021. “Rapid Adaptation Through Genomic and Epigenomic Responses Following Translocations in an Endangered Salmonid.” Evolutionary Applications 14, no. 10: 2470–2489. 10.1111/eve.13267. 34745338 PMC8549615

[eva70271-bib-0013] Elmer, K. R. , and A. Meyer . 2011. “Adaptation in the Age of Ecological Genomics: Insights From Parallelism and Convergence.” Trends in Ecology & Evolution 26, no. 6: 298–306. 10.1016/j.tree.2011.02.008. 21459472

[eva70271-bib-0014] Fadista, J. , A. K. Manning , J. C. Florez , and L. Groop . 2016. “The (In)famous GWAS *P*‐Value Threshold Revisted and Updated for Low‐Frequency Variants.” European Journal of Human Genetics 24: 1202–1205. 10.1038/ejhg.2015.269. 26733288 PMC4970684

[eva70271-bib-0016] Fausch, K. D. , Y. Taniguchi , S. Nakano , G. D. Grossman , and C. R. Townsend . 2001. “Flood Disturbance Regimes Influence Rainbow Trout Invasion Success Among Five Holarctic Regions.” Ecological Applications 11, no. 5: 1438–1455. 10.1890/1051-0761(2001)0115B1438:FDRIRT5D2.0CO;2.

[eva70271-bib-0015] Fausch, K. D. 2007. “Introduction, Establishment and Effects of Non‐Native Salmonids: Considering the Risk of Rainbow Trout Invasion in the United Kingdom.” Journal of Fish Biology 71: 1–32. 10.1111/j.1095-8649.2007.01682.x.

[eva70271-bib-0057] Garcia‐Erill, G. , and A. Albrechtsen . 2020. “Evaluation of model fit of inferred admixture proportions.” Molecular Ecology Resources 20, no. 4: 936–949. 10.1111/1755-0998.13171. 32323416

[eva70271-bib-0017] Gatz, A. J. , M. J. Sale , and J. M. Loar . 1987. “Habitat Shifts in Rainbow Trout: Competitive Influences of Brown Trout.” Oecologia 74: 7–19. 10.1007/BF00377339. 28310408

[eva70271-bib-0018] Goetz, L. C. , H. Nuetzel , D. L. J. Vendrami , et al. 2024. “Genetic Parentage Reveals the (Un)natural History of Central Valley Hatchery Steelhead.” Evolutionary Applications 17: e13681. 10.1111/eva.13681. 38516205 PMC10956469

[eva70271-bib-0019] Halverson, A. 2010. An Entirely Synthetic Fish: How Rainbow Trout Beguiled America and Overran the World. Yale University Press. 10.2307/j.ctt1nq8bk.

[eva70271-bib-0020] Hauser, L. , G. R. Carvalho , and T. J. Pitcher . 1995. “Morphological and Genetic Differentiation of the African Clupeid *Limnothrissa miodon* 34 Years After Its Introduction to Lake Kivu.” Journal of Fish Biology 47: 127–144. 10.1111/j.1095-8649.1995.tb06049.x.

[eva70271-bib-0021] Hayes, J. W. 1987. “Competition for Spawning Space Between Brown ( *Salmo trutta* ) and Rainbow Trout ( *S. gairdneri* ) in a Lake Inlet Tributary, New Zealand.” Canadian Journal of Fisheries and Aquatic Sciences 44, no. 1: 40–47. 10.1139/f87-005.

[eva70271-bib-0022] Kanno, Y. , M. A. Kulp , S. E. Moore , and G. D. Grossman . 2017. “Native Brook Trout and Invasive Rainbow Trout Respond Differently to Seasonal Weather Variation: Spawning Time Matters.” Freshwater Biology 62, no. 5: 868–879. 10.1111/fwb.12906.

[eva70271-bib-0023] Koene, J. P. , M. Crotti , K. R. Elmer , and C. E. Adams . 2019. “Differential Selection Pressures Result in a Rapid Divergence of Donor and Refuge Populations of a High Conservation Value Freshwater Fish *Coregonus lavaretus* (L.).” Evolutionary Ecology 33: 533–548. 10.1007/s10682-019-09995-y.

[eva70271-bib-0024] Korneliussen, T. S. , A. Albrechtsen , and R. Nielsen . 2014. “ANGSD: Analysis of Next Generation Sequencing Data.” BMC Bioinformatics 15: 356. 10.1186/s12859-014-0356-4. 25420514 PMC4248462

[eva70271-bib-0025] Koutsikos, N. , L. Vardakas , S. Zogaris , C. Perdikaris , O.‐I. Kalantzi , and A. N. Economou . 2019. “Does Rainbow Trout Justify Its High Rank Among Alien Invasive Species? Insights From a Nationwide Survey in Greece.” Aquatic Conservation: Marine and Freshwater Ecosystems 29, no. 3: 409–423. 10.1002/aqc.3025.

[eva70271-bib-0026] Landergren, P. 1999. “Spawning of Anadromous Rainbow Trout, *Oncorhynchus mykiss* (Walbaum): A Threat to Sea Trout, *Salmo trutta* L., Populations.” Fisheries Research 40, no. 1: 55–63. 10.1016/S0165-7836(98)00215-X.

[eva70271-bib-0027] Lázari, C. , C. Riva‐Rossi , J. Ciancio , et al. 2024. “Ancestry and Genetic Structure of Resident and Anadromous Rainbow Trout (*Oncorhynchus*) in Argentina.” Journal of Fish Biology 104, no. 6: 1972–1989. 10.1111/jfb.15722. 38556852

[eva70271-bib-0028] Li, H. 2013. “Aligning Sequence Reads, Clone Sequences and Assembly Contigs With BWA‐MEM. *arXiv*.” 10.48550/arXiv.1303.3997.

[eva70271-bib-0029] Lorenzen, K. , M. C. M. Beveridge , and M. Mangel . 2012. “Cultured Fish: Integrative Biology and Management of Domestication and Interactions With Wild Fish.” Biological Reviews 87, no. 3: 639–660. 10.1111/j.1469-18X.2011.00215.x. 22221879

[eva70271-bib-0030] Lou, R. N. , and N. O. Therkildsen . 2022. “Batch Effects in Population Genomic Studies With Low‐Coverage Whole Genome Sequencing Data: Causes, Detection and Mitigation.” Molecular Ecology Resources 22, no. 5: 1679–1692. 10.1111/1755-0998.13559. 34825778

[eva70271-bib-0031] Lowe, S. , M. Browne , S. Boudjelas , and M. De Porter . 2000. “100 of the World's Worst Invasive Alien Species: A Selection From the Global Invasive Species Database. ISSG/SSE/IUCN.” https://www.issg.org/booklet.pdf.

[eva70271-bib-0032] McGlade, C. L. O. , J. W. E. Dickey , R. Kennedy , et al. 2022. “Behavioural Traits of Rainbow Trout and Brown Trout May Help Explain Their Differing Invasion Success and Impacts.” Scientific Reports 12: 1757. 10.1038/s41598-022-05484-5. 35110590 PMC8810905

[eva70271-bib-0033] Meisner, J. , and A. Albrechtsen . 2018. “Inferring Population Structure and Admixture Proportions in Low‐Depth NGS Data.” Genetics 210, no. 2: 719–731. 10.1534/genetics.118.301336. 30131346 PMC6216594

[eva70271-bib-0034] Mueller, M. , L. Egg , T. Ruff , A. Haas , M. Schubert , and B. Gum . 2025. “Evidence of Natural Reproduction of North American Rainbow Trout ( *Oncorhynchus mykiss* ) in Three Alpine Rivers in Bavaria, Germany.” Fisheries Management and Ecology 32, no. 2: e12765. 10.1111/fme.12765.

[eva70271-bib-0035] Osmond, D. R. , R. A. King , I.‐R. M. Russo , M. W. Bruford , and J. R. Stevens . 2024. “Living in a Post‐Industrial Landscape: Repeated Patterns of Genetic Divergence in Brown Trut ( *Salmo trutta* L.) Across the British Isles.” Diversity and Distributions 30, no. 7: e13854. 10.1111/ddi.13854.

[eva70271-bib-0036] Paris, J. R. , R. A. King , and J. R. Stevens . 2015. “Human Activity Across the Ages Determines the Genetic Structure of Modern Brown Trout ( *Salmo trutta* L.) Populations.” Evolutionary Applications 8, no. 6: 573–585. 10.1111/eva.12266. 26136823 PMC4479513

[eva70271-bib-0037] Pearse, D. E. , N. J. Barson , T. Nome , et al. 2019. “Sex‐Dependent Dominance Maintains Migration Supergene in Rainbow Trout.” Nature Ecology & Evolution 3: 1731–1742. 10.1038/s41559-019-1044-6. 31768021

[eva70271-bib-0038] Pearse, D. E. , M. R. Miller , A. Abadía‐Cardoso , and J. C. Garza . 2014. “Rapid Parallel Evolution of Standing Variation in a Single, Complex, Genomic Region Is Associated With Life History in Steelhead/Rainbow Trout.” Proceedings of the Royal Society B 281, no. 1783: 20140012. 24671976 10.1098/rspb.2014.0012 PMC3996610

[eva70271-bib-0039] Petersson, E. , T. Jauvri , N. G. Steffner , and B. Ragnarsson . 1996. “The Effect of Domestication on Some Life History Traits of Sea Trout and Atlantic Salmon.” Journal of Fish Biology 48, no. 4: 776–791. 10.1111/j.1095-8649.1996.tb01471.x.

[eva70271-bib-0040] R Core Team . 2025. R: A Language and Environment for Statistical Computing. R Foundation for Statistical Computing. https://www.R‐project.org/.

[eva70271-bib-0041] Roman, J. 2006. “Diluting the Founder Effect: Cryptic Invasions Expand a Marine Invader's Range.” Proceedings of the Royal Society B 273, no. 1600: 2453–2459. 10.1098/rspb.2006.3597. 16959635 PMC1634897

[eva70271-bib-0042] Ros, A. , H. Schmidt‐Posthaus , and A. Brinker . 2022. “Mitigating Human Impacts Including Climate Change on Proliferative Kidney Disease in Salmonids of Running Waters.” Journal of Fish Diseases 45: 497–521. 10.1111/jfd.13585. 35100455

[eva70271-bib-0043] Rubin, A. , C. Bailey , N. Strepparava , T. Wahli , H. Segner , and J.‐F. Rubin . 2022. “Reliable Field Assessment of Proliferative Kidney Disease in Wild Brown Trout, *Salmo trutta* , Populations: When Is the Optimal Sampling Period?” Pathogens 11, no. 6: 681. 10.3390/pathogens11060681. 35745535 PMC9230507

[eva70271-bib-0044] Schluter, D. 2009. “Evidence for Ecological Speciation and Its Alternative.” Science 323, no. 5915: 737–741. 10.1126/science.1160006. 19197053

[eva70271-bib-0045] Shafer, A. B. A. , C. R. Peart , S. Tusso , et al. 2017. “Bioinformatic Processing of RAD‐Seq Data Dramatically Impacts Downstream Population Genetic Inference.” Methods in Ecology and Evolution 8: 907–917. 10.1111/2041-210X.12700.

[eva70271-bib-0046] Simberloff, D. 2009. “The Role of Propagule Pressure in Biological Invasions.” Annual Review of Ecology, Evolution, and Systematics 40: 81–102. 10.1146/annurev.ecolsys.110308.120304.

[eva70271-bib-0047] Stanković, D. , A. J. Crivelli , and A. Snoj . 2015. “Rainbow Trout in Europe: Introduction, Naturalisation, and Impacts.” Reviews in Fisheries Science & Aquaculture 23, no. 1: 39–71. 10.1080/23308249.2015.1024825.

[eva70271-bib-0048] Stanković, D. , M. R. Stephens , and A. Snoj . 2016. “Origin and Introduction History of Self‐Sustaining Rainbow Trout Populations in Europe as Inferred From Mitochondrial DNA and a Y‐Linked Marker.” Hydrobiologia 770: 129–144. 10.1007/s10750-015-2577-6.

[eva70271-bib-0049] Tarasov, A. , A. J. Vilella , E. Cuppen , I. J. Mijman , and P. Prins . 2015. “Sambamba: Fast Processing of NGS Alignment Formats.” Bioinformatics 31, no. 12: 2032–2034. 10.1093/bioinformatics/btv098. 25697820 PMC4765878

[eva70271-bib-0050] Vuorinen, J. , T. F. Næsje , and O. T. Sandlund . 1991. “Genetic Changes in a Vendace *Coregonus albula* (L.) Population 92 Years After Introduction.” Journal of Fish Biology 39: 193–201. 10.1111/j.1095-8649.1991.tb05083.x.

[eva70271-bib-0051] Weber, G. M. , K. E. Martin , Y. Palti , S. Liu , J. N. Beach , and J. E. Birkett . 2023. “Effects of Fertilizing Eggs From a Summer‐Spawning Line With Cryopreserved Milt From a Winter‐Spawning Line on Spawning Date and Egg Production Traits in Rainbow Trout.” Aquaculture Reports 29: 101495. 10.1016/j.aqrep.2023.101495.

[eva70271-bib-0052] Weeder, J. A. , A. R. Marshall , and J. M. Epifanio . 2005. “An Assessment of Population Genetic Variation in Chinook Salmon From Seven Michigan Rivers 30 Years After Introduction.” North American Journal of Fisheries Management 25, no. 3: 861–875. 10.1577/M03-227.1.

[eva70271-bib-0053] Weinstein, S. Y. , F. P. Thrower , K. M. Nichols , and M. C. Hale . 2019. “A Large‐Scale Chromosomal Inversion Is Not Associated With Life History Development in Rainbow Trout From Southeast Alaska.” PLoS One 14, no. 9: e0223018. 10.1371/journal.pone-0223018. 31539414 PMC6754156

[eva70271-bib-0054] Yin, L. , H. Zhang , Z. Tang , et al. 2021. “rMVP: A Memory‐Efficient, Visualization‐Enhanced, and Parallel‐Accelerated Tool for Genome‐Wide Association Study.” Genomics, Proteomics & Bioinformatics 19, no. 4: 619–628. 10.1016/j.gpb.2020.10.007. PMC9040015 33662620

[eva70271-bib-0055] Young, K. A. , J. B. Dunham , J. F. Stephenson , et al. 2010. “A Trial of Two Trouts: Comparing the Impacts of Rainbow and Brown Trout on a Native Galaxiid.” Animal Conservation 13, no. 4: 399–410. 10.1111/j.1469-1795.2010.00354.x.

[eva70271-bib-0056] Zhou, X. , and M. Stephens . 2012. “Genome‐Wide Efficient Mixed‐Model Analysis for Association Studies.” Nature Genetics 44: 821–824. 10.1038/ng.2310. 22706312 PMC3386377

