# Supplementary material for: Multiple Genetic Origins of Non‐Native, Self‐Sustaining Rainbow Trout Oncorhynchus mykiss in Streams in Baden‐Württemberg, Germany
Source: Evol Appl. 2026 May 24;19(5):e70271. doi: 10.1111/eva.70271 (PMC13239981; doi:10.1111/eva.70271)
Supplement: Supplementary file 1 — Figure S1: Admixture analysis across 3 hatchery and 14 wild stream populations of rainbow trout. Genetic structure was best explained with 15 genetic clusters (K = 15), based on the number of principal components in PCAngsd and the correlation of residuals (vide Figure S2, below). *Nb. Breg is represented by a single individual. Figure S2:. EvalAdmix results. Shown are correlations of residuals for each pairwise comparison of individuals/populations. Correlations were inferred with evalAdmix for ancestry proportions in Figure S1 and were used to select the value of K at which correlations of residuals are minimised (closest to 0) (K = 15). Cells above the diagonal are individual‐specific, and cells below the diagonal are averaged by population. Population and ecotype labels are shown next to each group. *Nb. Breg is represented by a single individual. Figure S3:. Genetic diversity measured as π in 100 kb windows across the genome of 3 hatchery and 13 stream populations of rainbow trout. One stream population (Breg) was omitted because it was represented by a single individual. Outliers are included in (A), and excluded in (B). Figure S4:. Genetic diversity measured as Watterson's θ in 100 kb windows across the genome of 3 hatchery and 13 stream populations of rainbow trout. One stream population (Breg) was omitted because it was represented by a single individual. Outliers are included in (A), and excluded in (B). Figure S5:. Pairwise weighted FST heatmap of 120 comparisons across 16 populations of rainbow trout. Dashed lines separate hatchery (red) from wild stream populations. Figure S6:. Genome‐wide differentiation between hatchery and stream populations in 100 kb sliding windows and 25 kb steps. Slightly broader peaks of differentiation (e.g., on chr28) also show similar signals in the SNP‐by‐SNP comparison (Figure 4A). [file EVA-19-e70271-s001.docx]

**Supplementary material**

**Figure S1.** Admixture analysis across 3 hatchery and 14 wild stream populations of rainbow trout. Genetic structure was best explained with 15 genetic clusters (K = 15), based on the number of principal components in *PCAngsd* and the correlation of residuals (*vide* Fig. S2, below). * Nb. Breg is represented by a single individual.

**Figure S2.** EvalAdmix results. Shown are correlations of residuals for each pairwise comparison of individuals/populations. Correlations were inferred with evalAdmix for ancestry proportions in Figure S1 and were used to select the value of K at which correlations of residuals are minimised (closest to 0) (K=15). Cells above the diagonal are individual-specific, and cells below the diagonal are averaged by population. Population and ecotype labels are shown next to each group. * Nb. Breg is represented by a single individual.

**Figure S3.** Genetic diversity measured as *π* in 100 kb windows across the genome of 3 hatchery and 13 stream populations of rainbow trout. One stream population (Breg) was omitted because it was represented by a single individual. Outliers are included in (A), and excluded in (B).

**Figure S4.** Genetic diversity measured as Watterson’s *θ* in 100 kb windows across the genome of 3 hatchery and 13 stream populations of rainbow trout. One stream population (Breg) was omitted because it was represented by a single individual. Outliers are included in (A), and excluded in (B).


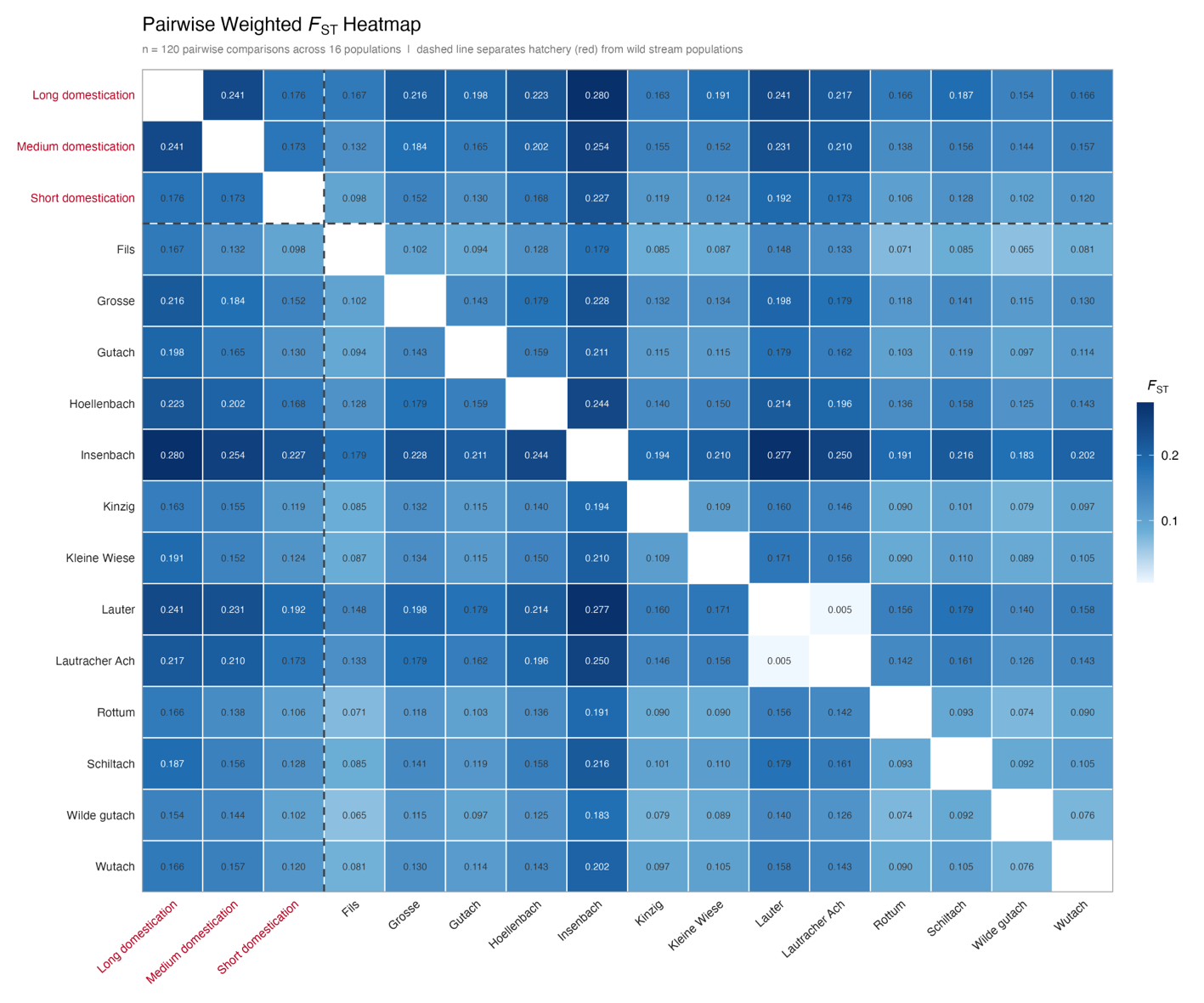


**Figure S5.** Pairwise weighted F_ST_ heatmap of 120 comparisons across 16 populations of rainbow trout. Dashed lines separate hatchery (red) from wild stream populations.


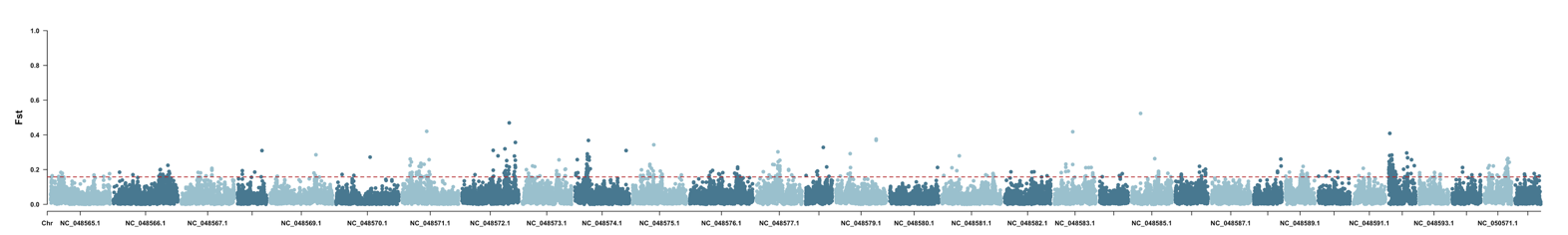


**Figure S6.** Genome-wide differentiation between hatchery and stream populations in 100kb sliding windows and 25kb steps. Slightly broader peaks of differentiation (e.g. on chr28) also show similar signals in the SNP-by-SNP comparison (Fig. 4A).
